# Changes in Locomotor Ratio During Basketball Game Quarters From Elite Under-18 Teams

**DOI:** 10.3389/fpsyg.2019.02163

**Published:** 2019-09-20

**Authors:** Jairo Vázquez-Guerrero, Bruno Fernández-Valdés, Bruno Gonçalves, Jaime E. Sampaio

**Affiliations:** ^1^Sport Performance Department, FC Barcelona Sports, Barcelona, Spain; ^2^National Institute of Physical Education of Catalonia (INEFC), Barcelona, Spain; ^3^Research Center in Sports Sciences, Health Sciences and Human Development, CIDESD, CreativeLab Research Community, Vila Real, Portugal

**Keywords:** physical performance, competition demands, workload, playing positions, accelerometry, fatigue

## Abstract

Quantifying game and training demands in basketball allows to determine player’s readiness and optimizes preparation to perform and reduce injury risks. Available research is using tracking technology to perform general descriptions of the game activities at professional levels, but somehow, is not exploring the possibilities of gathering data from new variables that can contribute with complementary information for the coaching staffs. The aim of this study was to identify changes in locomotor ratio, at higher and lower speeds, during the game quarters from elite under-18 basketball teams. Ninety-four male players participated in the study (age: 17.4 ± 0.74 years; height: 199.0 ± 0.1 cm; body mass: 87.1 ± 13.1 kg) from different playing positions, Guards (*n* = 35), Forwards (*n* = 42), and Centers (*n* = 17). Data were gathered from an international tournament and players’ movements were measured using a portable ultra-wide band position-tracking system (WIMU PRO^®^, Realtrack Systems, Almeria, Spain). The following variables were measured: (1) relative distance in different speed zones: walking (<6.0 km·h^−1^), jogging (6.0–12.0 km·h^−1^), running (12.1–18.0 km·h^−1^), high-intensity running (18.1–24.0 km·h^−1^), and sprinting (>24.1 km·h^−1^); and (2) player load, vector magnitude expressed as the square root of the sum of the squared instantaneous rates of change in acceleration in each of the three planes divided by 100. Afterward, these variables were used to calculate players’ locomotor ratio (player load per meter covered) at higher (running, high-intensity running, and sprinting) and lower speeds (walking and jogging). Results from the locomotor ratio at both lower and higher speeds presented a significant effect for the quarter (*F* = 7.3, *p* < 0.001 and *F* = 7.1, *p* < 0.001, respectively) and player position (*F* = 3.1, *p* = 0.04, *F* = 9.2, *p* < 0.001, respectively). There was an increase in the locomotor ratio from game quarter (Q) Q1 to Q4 at lower speeds, but contrary trends at higher speeds, i.e., the values have decreased from Q1 to Q4. Also, forwards and centers of the best teams presented lower values at higher speeds. Altogether, the findings may be used by coaching staffs as a first baseline to elaborate normative behavior models from the players’ performance and also to induce variability and adaptation in specific practice planning.

## Introduction

Understanding team sports training and competition effects is currently a hot topic in sports medicine and sports sciences (Soligard et al., 2016), with particular relevance to the professional work of strength and conditioning coaches. The process of monitoring load is particularly important as it helps to quantify training and game demands, in order to improve the coaching decisions regarding periodization plans and administration of training loads (Rogalski et al., 2013). All previous research were relatively consensual suggesting that mistakes that result in under or overloading the players, frequently lead to substantial decreases in performance and can increase injury occurrences (Orchard, 2012; Caparros et al., 2016).

Sports scientists frequently obtain data from the external training load associated with the correspondent internal load responses (Soligard et al., 2016; Akubat et al., 2018; McLaren et al., 2018; Impellizzeri et al., 2019; Oliveira et al., 2019), however, acquiring data from the internal load (such as blood lactate or heart rate) during official competitions can be unrealistic and frequently very restricted. The studies focused on the relationships between internal and external loads in team sports report correlation magnitudes ranging from trivial to very large, suggesting that those relationships are not yet fully understood (McLaren et al., 2018). Therefore, tracking team sports activities with the most appropriate methods and variables can be one of the major challenges in contemporary research (Soligard et al., 2016; Oliveira et al., 2019).

There is a wide range of variables collected and processed that basically include measures of distances covered at different speed zones, accelerations, decelerations, jumps, or impacts (Bangsbo et al., 1991; Andrzejewski et al., 2018; Rago et al., 2019). The studies using these variables have allowed to understand better the players’ responses to training and competition. For example, in association football, recent research has reported that high-speed running and total distance covered decreased during the last phases of each half (Weston et al., 2011) and in the last 15 min of professional match-play (Russell et al., 2016). In addition, there is a higher incidence of injuries over time in both the first and second halves either in professional (Hawkins et al., 2001; Woods et al., 2004; Ekstrand et al., 2011) and in youth players (Price et al., 2004). Concretely, the greatest injury rate has been shown to occur in the last 15 min of each half, especially in the second part of the match (Hawkins et al., 2001; Woods et al., 2004), may be due to fatigue (Greig and McNaughton, 2014; McCall et al., 2014).

More recently, research in association football has described and presented a new variable designated as locomotor efficiency, calculated using the ratio between player load (obtained with tri-axial accelerometer data) and locomotor activities such as total distance covered or high-speed running distances (Barrett et al., 2016). This study concluded that locomotor efficiency may be a useful variable to identify fatigue and increases in injury risk, by identifying reductions in three-dimensional loading, a trend synonymous of greater injury incidence. The authors calculated the ratio of player load to total distance covered as a potential measure of locomotor efficiency, attempting to identify uncoupling moments, which may be indicative of player fatigue (Barrett et al., 2016).

In basketball, using the training monitoring process to help informing the coaching staffs, and presumably, help preventing injuries is a very relevant issue (Deitch et al., 2006). In fact, the injuries have a serious effect on team performance (Caparros et al., 2018), as seen by the relevant number of matches that players are unavailable to participate (Podlog et al., 2015; Caparros et al., 2016). However, the available research is very scarce and reports contradictions. For example, no differences have been reported in locomotor movement patterns through the match time in elite under-19-year-old basketball players (McInnes et al., 1995), as shown by the percent of live time spent in high-intensity locomotor actions that decreased from 17.58 ± 1.76% during the first quarter to 13.64 ± 1.33% in the last quarter. However, other study carried in junior elite basketball, suggested that basketball players suffered from fatigue effects as the game time advanced (Ben Abdelkrim et al., 2010). Concretely, compared with the first half, the distance covered by sprinting and striding running decreased significantly in the second half (411 ± 101 vs. 352 ± 97 m; 222 ± 73 vs. 185 ± 45 m, respectively), whereas distance covered by walking and jogging increased (818 ± 88 vs. 901 ± 114 m; 886 ± 167 vs. 984 ± 189 m, respectively).

A recent review focused on the activity demands and physiological responses during basketball match-play has summed up 25 articles and concluded that during live playing time across 40-min matches, male and female basketball players traveled 5–6 km at an average physiological intensity above the lactate threshold and 85% HRmax (Stojanovic et al., 2018). It also became clear that research in basketball is still limited to the analysis of the same variables, due to the lack of technology capable of collecting reliable locomotor data in indoor courts. Very recently, this technology has been allowing to expand the topic and improve the descriptions from the game activities and training sessions (Schelling and Torres, 2016; Paulauskas et al., 2019; Svilar et al., 2019). However, there is still a clear need to investigate deeper how different variables, such as the locomotor ratio, can provide more and relevant information to the coaching staffs. Accordingly, the aim of this study was to identify changes in the locomotor ratio, at several speeds, during the game quarters in U-18 basketball games. Knowing these changes during the basketball games can help the coaching staffs and strength and conditioning trainers to design more adequate training sessions and conditioning programs that aim to improve players’ fitness and minimize fatigue and injury risk factors.

## Materials and Methods

### Participants

The subjects were part of eight teams from six countries professional men’s basketball teams, competing in the Euroleague Basketball Next Generation Tournament in Istanbul, Turkey. Ninety-four male subjects participated in the study (age: 17.4 ± 0.7 years; height: 199.0 ± 0.1 cm; body mass: 87.1 ± 13.1 kg) from different playing positions, Guards (*n* = 35), Forwards (*n* = 42), and Centers (*n* = 17). The Tournament was played from 18 to 21 May 2017. The teams were asked for permission to be monitored and all the players and coaches were informed about the research protocol, requirements, benefits, and risks, and their consent was obtained before the study began. All the teams had access to a performance report following the games. The study protocol was approved and followed the guidelines stated by the Ethics Committee of the University of Trás-os-Montes and Alto Douro, based at Vila Real (Portugal) and conformed to the recommendations of the Declaration of Helsinki.

### Design

A non-experimental descriptive design was used to examine the differences between physical demands for playing positions during the official competitive games from the tournament. The players’ physical activity was assessed using the WIMU PRO^®^ UWB-based position-tracking systems System (Realtrack Systems, Almeria, Spain) (Bastida Castillo et al., 2018). The devices were installed around the basketball court, with four antennae located at 4.5 m from the corners of the field and two antennae located at 5.5 m of the field, forming a hexagon for a better emission and reception of the signal. All games were played based on FIBA rules, started with a 15-min warm-up based on ball dribbling, shooting, specific mobility, and dynamic stretching exercises, and were performed on the same court in similar environmental conditions. Players were able to replace water loss by drinking *ad libitum* during recovery periods. A total of 13 games were accessed over the 4-day tournament, all teams played three games, exception made to the two finalist teams that played four games. During each game, all the players were continuously monitored and the database included a total of 769 cases. The physical demands were quantified only when players were competing on the court (e.g., removing periods when a player was a substitute, or when there was a rest time between quarters).

### Physical Demands

Players’ movements were measured using a portable UWB-based position-tracking system (WIMU PRO^®^, Realtrack Systems SL, Almería, Spain) during games. These devices (81 mm × 45 mm× 15 mm, 70 g) were fitted to the upper back of each player using an adjustable harness (Rasán, Valencia, Spain). The units integrate different sensors, registering at different sample frequencies. Sampling frequency for 3-axis accelerometer, gyroscope, and magnetometer was 100 Hz and 120 kPa for the barometer. The system has six ultra-wideband antennas, four of them placed 3 m outside the corners of the court and two placed 3 m outside half-court, the sampling frequency for positioning data were 20 Hz. The system operates using triangulation between the antennas and the units, the six antennas send a signal to the units every 50 ms. Then, the device calculates the time required to receive the signal and derives the unit position (coordinates X and Y), using one of the antennas as a reference. Data were analyzed using the system-specific software (SPRO Software, Realtrack Systems SL, Almería, Spain). The system has been showing good validity with better accuracy (bias: 0.57–5.85%), test-retest reliability (%TEM: 1.19), and inter-unit reliability (bias: 0.18) in determining distance covered compared to GPS technology (bias: 0.69–6.05%; %TEM: 1.47; bias: 0.25). Also, it showed better results (bias: 0.09; ICC: 0.979; bias: 0.01) for mean velocity measurement than GPS (bias: 0.18; ICC: 0.951; bias: 0.03) (Bastida Castillo et al., 2018). Nevertheless, to confirm calibration and prevent any errors due to excessive usage, the reliability of the system was re-inspected during the study period using line and v-cut movements while walking, jogging, running, and sprinting. The average values of the intraclass-correlation between obtained distances and real distances were very high (ICC = 0.973, 95% CI ranged between 0.964 and 0.980) and the coefficient of variation from these differences was low (CV = 1.3%).

The following variables were measured: (1) relative distance in established speed zones (McInnes et al., 1995; Puente et al., 2017): stationary/walking (<6.0 km·h^−1^), jogging (6.0–12.0 km·h^−1^), running (12.1–18.0 km·h^−1^), high-intensity running (18.1–24.0 km·h^−1^), and sprinting (>24.1 km·h^−1^); and (2) player load, vector magnitude expressed as the square root of the sum of the squared instantaneous rates of change in acceleration in each of the three planes divided by 100 (Hawkins et al., 2001; Woods et al., 2004). These variables were used to calculate players’ locomotor ratio (player load per meter covered) at higher (running, high-intensity running, and sprinting) and lower speeds (walking and jogging) (Barrett et al., 2016). The relative locomotor ratio was calculated by normalizing the highest score to 100% and the lowest to 0%.

### Statistical Analyses

Descriptive statistics (mean ± SD) for the outcome measures were calculated. A linear mixed effects model was used to model the main and interactive effects of the game quarter (Q1, Q2, Q3, or Q4) and playing the position (Guards, Forwards, and Centers) were treated as the fixed effects, whereas the random effects were player ID and match code. The two locomotor ratio variables were used throughout as the dependent variables. The analysis was performed using the Statistical Package for the Social Sciences software (SPSS Inc., Chicago, IL), and statistical significance was set at *p* < 0.05. The *post hoc* comparisons were carried through estimation inferences. The comparisons between quarters were assessed *via* standardized mean differences and respective 90% confidence intervals. Thresholds for effect sizes statistics were 0.2, trivial; 0.6, small; 1.2, moderate; 2.0, large; and > 2.0, very large (Hopkins, 2000).

## Results

**Figure 1:**
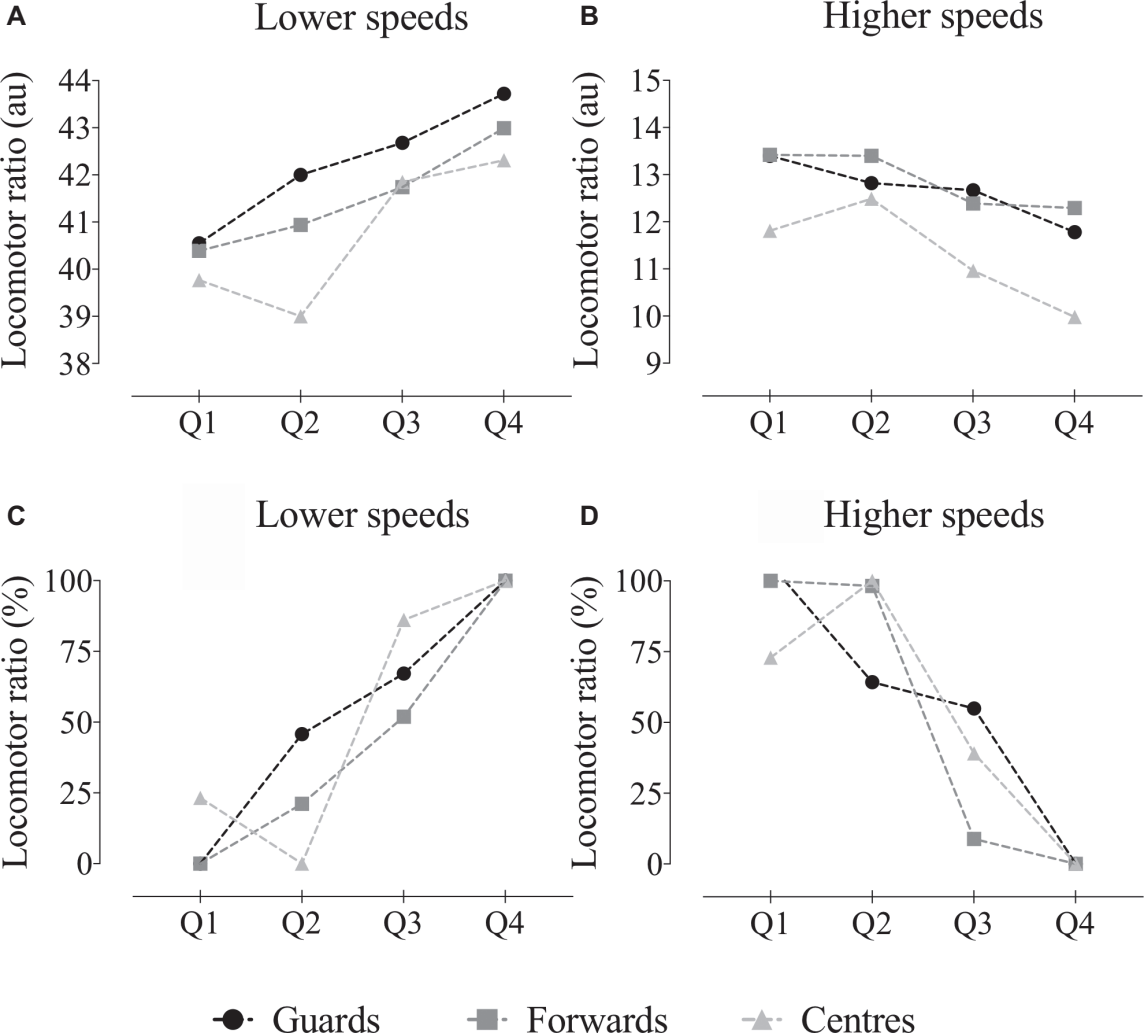
Absolute (au) and relative locomotor ratio (%) at lower **(A,C)** and higher speeds **(B,D)**, across game quarters for guards, forwards, and centers. Lower speeds: significant main effects of quarter and position; higher speeds: significant main effects of quarter and position;

Results from the locomotor ratio at lower speeds presented a significant effect for the quarter (*F* = 7.3, *p* < 0.001) and position (*F* = 3.1, *p* = 0.04), but not for the interaction (*F* = 0.4, *p* = 0.86, see Figures 1A,C). It was possible to identify increases in the locomotor ratio from Q1 to Q4, which was similar for all playing positions. The guards had a higher locomotor ratio at lower speeds. At higher speeds, the locomotor ratio presented a contrary trend. Results showed a significant effect for the quarter (*F* = 7.1, *p* < 0.001) and position (*F* = 9.2, *p* < 0.001), but not for the interaction (*F* = 0.7, *p* = 0.63, see Figures 1B,D). However, there was a decrease in the locomotor ratio from Q1 to Q4, similar in all playing positions. The centers had lower a locomotor ratio at higher speeds.

**Figure 2:**
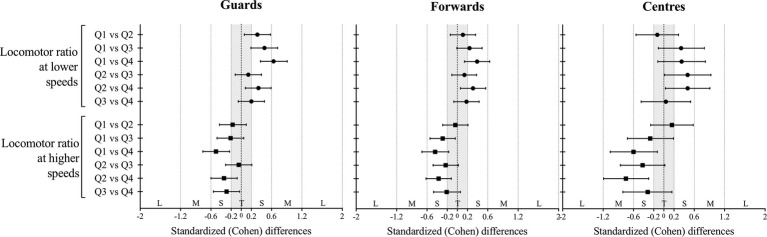
Standardized (Cohen) differences in locomotor ratio outcomes according to the game quarter for each playing positions. Error bars indicate uncertainty in the true mean changes with 90% confidence intervals.

Figure 2 depicts the standardized differences between the levels of the studied factors. Higher magnitudes were observed between Q1 and Q4 with contrary trends for lower and higher speeds. The guards’ position increased 7.7%; ±3.3% (mean difference with 90% confidence limits) at lower speeds and decreased −12.3%; ±6.1% at higher (moderate and small effect, respectively) from first to the fourth quarter. In the same line, forwards increased 6.4%; ±4.2% at lower speeds and decreased −11.5%; ±6.6% higher (both small effects). Results from the centers present more variability.

**Figure 3:**
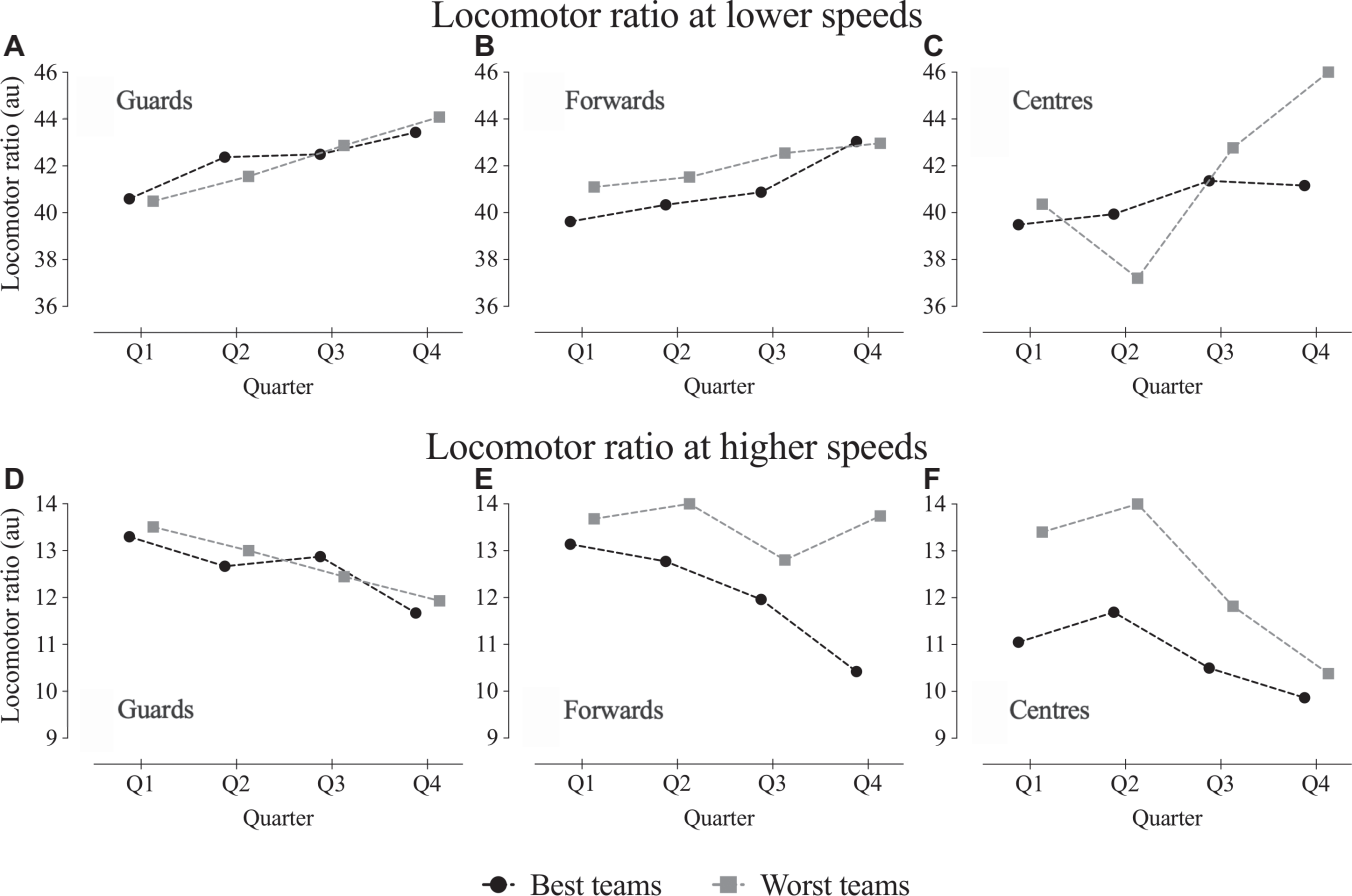
Locomotor ratio (au) at lower and higher speeds (lower and upper panel, respectively) across game quarters for guards **(A,D)**, forwards **(B,C)**, and centers **(C,F)**, and separated by the best and worst teams. Lower speeds: no differences found; higher speeds: significant main effects of team quality and interaction team quality × position.

Figure 3 shows the locomotor ratio when using the team quality as criteria (best and worst teams). Results from locomotor ratio at lower speeds showed no significant effect of interactions with team quality. At higher speeds, there were significant effects of team quality (*F* = 13.8, *p* < 0.001) and there was a significant interaction team quality × position (*F* = 4.4, *p* = 0.013). In general, best teams had lower locomotor ratio scores, but this was particularly noted in forwards and centers.

## Discussion

The aim of this study was to identify changes in locomotor ratio, at several speeds, during the game quarters in U-18 basketball games. General key findings showed an increase in the locomotor ratio from Q1 to Q4 at lower speeds, but contrary trends at higher speeds, i.e., the locomotor ratio decreased from Q1 to Q4. Also, forwards and centers of the best teams presented lower values at higher speeds.

The locomotor ratio has been presented and computed by the ratio between player load and total distance covered, where an increase in output is suggestive of an increase in the loading required for every given meter of distance covered on the pitch (Barrett et al., 2016). Furthermore, the established links within match movements patterns, fatigue increase and injury incident (Barrett et al., 2016), highlighting its use as a useful and promising monitoring tool in team sports. However, the onset of fatigue is likely dependent on the physical and physiological demand required by the game (Ziv and Lidor, 2009) and therefore, by using the volume of the external load as the sole parameter, the specific reference to the most demanding physical requirements are not considered. With this limitation in mind, this study extended the interpretation and applicability of this variable by calculating the locomotor ratio at different speed displacements.

Decreases in activity demands have been systematically identified toward the end of basketball games, likely underpinned by fatigue-related mechanisms and tactical strategies (Stojanovic et al., 2018). In fact, the accumulated activity workload is well related to the tactical performances changes, particularly at faster speeds, where the players’ positioning is more predictable (Sampaio et al., 2014). Also, these game behavior patterns are strongly influenced by different playing levels, playing positions or competition type, whereby each position sustains different profiles in different performance indicators (Klusemann et al., 2013; Stojanovic et al., 2018). For example, a study carried with elite under-19-year-old basketball players showed that the first and third quarters were played at higher intensities than the second and fourth quarters and, that impaired physical performance occurs during the second and fourth quarters for players in all positions (Ben Abdelkrim et al., 2007). In the current study, the locomotor ratio increased from Q1 to Q4 at lower speeds, however, decreased at higher speeds. The progressive reduction in highly demanding loads likely reflects the typical within game time-motion patterns related to game context. The teams are likely to manage a time-extended control of ball possession and consequently, the proportion of straight play and fast breaks decreases, leading the game pace to slow down (Ben Abdelkrim et al., 2007). Also, the increase in mental fatigue and perceived exertion may have an important role since the impairment in endurance may be related with these indicators (Van Cutsem et al., 2017; Coutinho et al., 2018). However, further research is necessary to understand the mechanisms underlying performance impairments under this scope (Smith et al., 2018).

Overall, best teams presented lower locomotor ratio at higher speeds across all quarters. In fact, previous research has shown, with consistent results that optimized attention processes, which are key for perceiving the environmental information are related to expertise (Weast et al., 2011; Sampaio et al., 2015). Therefore, it is possible that better game decisions are also a consequence of constant positioning and the attune between teammates and opponents’ displacements, which may allow players to decrease the required efforts to perform. In fact, recent research in football association shows that higher levels of positional synchronization correspondent to lower levels of distance covered and heart rate responses, mostly in experienced players (Folgado et al., 2018). Regarding the playing positions, guards were the players that showed fewer differences at both lower and higher speeds when comparing best and worst teams. Actually, it has been reported that this position exhibits relatively lower game-to-game variability in technical and physical variables in comparison with players from other positions, and more specifically, trivial changes were found when comparing stronger vs. weaker guards (Zhang et al., 2017). Also, it has been shown that centers from weaker teams run more distance and at higher speeds (Zhang et al., 2017), which demands higher locomotor patterns of movements. Altogether, these findings may be used by coaching staffs as a first baseline to elaborate normative behavior models from the players’ performance and also to induce variability and adaptation in practice planning. The current study has only analyzed a congested period of competition in which players can accumulate fatigue. Further research can expand the current findings by revealing how these players’ responses can vary across a basketball season and how the individual fitness levels can affect these responses.

## Conclusion

This study showed an increase in the locomotor ratio from Q1 to Q4 at lower speeds, but contrary trends at higher speeds, i.e., the locomotor ratio has decreased from Q1 to Q4. Also, forwards and centers of the best teams presented lower values at higher speeds. The findings may be used by coaching staffs as a first baseline to elaborate normative behavior models from the players’ performance and also to induce variability and adaptation in specific practice planning.

## Data Availability Statement

The datasets generated for this study are available on request to the corresponding author.

## Ethics Statement

The study protocol was approved and followed the guidelines stated by the Ethics Committee of the University of Trás-os-Montes and Alto Douro, based ate Vila Real (Portugal) and conformed to the recommendations of the Declaration of Helsinki.

## Author Contributions

JV-G contributed in conceptualization. JV-G, BF-V, BG, and JS performed methodology. JV-G, BG, and JS conducted formal analysis. JV-G, BF-V, and JS helped in writing and preparing the original draft. JV-G, BF-V, BG, and JS contributed in writing, reviewing, and editing.

### Conflict of Interest

JV-G was employed by company FC Barcelona Sports.

The remaining authors declare that the research was conducted in the absence of any commercial or financial relationships that could be construed as a potential conflict of interest.
